# Supplementary material for: Systemic short chain fatty acids limit antitumor effect of CTLA-4 blockade in hosts with cancer
Source: Nat Commun. 2020 May 1;11:2168. doi: 10.1038/s41467-020-16079-x (PMC7195489; doi:10.1038/s41467-020-16079-x)
Supplement: Supplementary file 1 — Supplementary Information [file 41467_2020_16079_MOESM1_ESM.pdf]

Systemic Short Chain Fatty Acids limit antitumor effect of CTLA-4 blockade in hosts with cancer

C. Coutzac et al.

Supplementary Information

Supplementary Table 1-4

Supplementary Figures 1-23

Supplementary Tables

Supplementary Table 1. Clinical characteristics of patients treated with ipilimumab in two independent cohorts.

Characteristics	French (n=50)	Italian (n=45)
Median age – year [range]	64 [36-85]	66 [22-86]
Male sex (%)	27 (54)	31 (62)
**Immune checkpoint-blockade indication, n (%)**		
Melanoma	48 (96)	45 (100)
Prostate carcinoma	2 (4)	0 (0)
Melanoma stage *, n (%)		
M1a	12 (24)	7 (16)
M1b	2 (4)	6 (13)
M1c	36 (72)	32 (71)
**Prior therapies, n (%)**		
None	31 (62)	30 (67)
Chemotherapy	5 (10)	3 (7)
BRAF-inhibitor	2 (4)	9 (20)
Hormonotherapy	2 (4)	0
Immune check point inhibitor	7 (14)	1 (2)
Others	3 (8)	2 (4)
**Treatment after progression, n (%)**		
None	20 (40)	9 (20)
Anti-PD-1	21 (42)	28 (62)
Others	9 (18)	8 (18)
Ipilimumab induced colitis (%)	10 (20)	9 (20)
Median survival - month [range]	18 [1-35]	12 [2-36]
Median progression - month [range]	4 [1-35]	3 [1-32]

Supplementary Table 2. French cohort samples collection. Samples collected for each patient for serum, fresh whole blood (WB) and stools at V_1 (baseline i.e. before ipilimumab introduction), V_2 (before the second injection of ipilimumab) and V_3 (before the third injection of ipilimumab) are indicated. YES = Analysis performed on this sample; NS = No Sample; ND** = Not enough aliquots to perform all analyses. YES£= for patient #18 CCR7 flow cytometry antibody was missing in the tube thus memory T cells was not available at V3, for patient #26 ICOS flow cytometry antibody was missing in the tube thus ICOS positive T cells were not available at V1 for this patient.

Patients #	Stools Q-PCR/NGS	SCFA stools (V1)	SCFA serum (V1)	Serum markers (V1)	Serum markers (V2)	Serum markers (V3)	WB (V1)	WB (V2)	WB (V3)
1	YES	ND**	YES	YES	YES	YES	YES	YES	NS
2	YES	YES	YES	YES	YES	YES	YES	YES	YES
3	YES	YES	YES	YES	YES	YES	YES	YES	YES
4	NS	NS	NS	YES	YES	NS	YES	YES	NS
5	YES	YES	YES	YES	YES	YES	YES	YES	YES
6	YES	YES	YES	YES	YES	YES	YES	YES	YES
7	YES	YES	YES	YES	YES	YES	YES	YES	YES
8	NS	NS	NS	YES	YES	YES	YES	YES	YES
9	YES	YES	YES	YES	YES	YES	YES	YES	YES
10	YES	YES	YES	YES	NS	NS	YES	NS	NS
11	YES	YES	YES	YES	NS	YES	YES	YES	NS
12	YES	YES	YES	YES	YES	NS	YES	YES	NS
13	YES	YES	YES	YES	YES	YES	YES	YES	YES
14	NS	NS	NS	YES	YES	NS	YES	YES	NS
15	YES	YES	YES	YES	YES	YES	YES	YES	YES
16	YES	YES	NS	NS	NS	NS	NS	YES	YES
17	YES	YES	YES	YES	YES	YES	YES	YES	YES
18	ND**	YES	YES	ND**	NS	NS	YES	NS	YES£
19	YES	YES	YES	YES	YES	NS	YES	YES	NS
20	NS	NS	NS	YES	YES	YES	YES	YES	YES
21	YES	YES	YES	YES	YES	YES	YES	YES	YES
22	ND**	YES	YES	YES	YES	NS	YES	YES	NS
23	YES	YES	YES	YES	YES	YES	YES	YES	YES
24	YES	YES	NS	NS	YES	NS	NS	YES	NS
25	YES	YES	NS	NS	NS	NS	NS	NS	YES
26	YES	YES	YES	YES	YES	YES	YES£	YES	YES
27	YES	YES	YES	YES	YES	YES	YES	YES	YES
28	YES	YES	YES	YES	YES	YES	YES	YES	YES
29	NS	NS	YES	ND**	NS	NS	NS	YES	YES
30	YES	YES	YES	ND**	NS	NS	NS	YES	YES
31	YES	YES	YES	YES	YES	YES	YES	YES	YES
32	NS	NS	NS	NS	NS	NS	NS	NS	NS
33	YES	YES	YES	YES	YES	YES	YES	YES	YES
34	NS	NS	YES	YES	YES	YES	YES	YES	YES
35	YES	YES	YES	YES	YES	YES	YES	YES	YES
36	NS	NS	YES	YES	YES	YES	YES	YES	YES
37	YES	YES	YES	YES	YES	YES	YES	YES	YES
38	ND**	YES	YES	YES	YES	NS	YES	NS	NS
39	YES	YES	YES	YES	YES	YES	YES	YES	YES
40	YES	YES	YES	YES	YES	YES	YES	YES	YES
41	YES	YES	YES	YES	YES	YES	YES	YES	YES
42	YES	YES	NS	NS	NS	NS	YES	YES	YES
43	YES	YES	YES	ND**	NS	NS	YES	YES	NS
44	YES	YES	YES	ND**	NS	NS	YES	YES	YES
45	YES	YES	YES	ND**	NS	NS	YES	NS	YES
46	YES	YES	YES	ND**	NS	NS	YES	YES	YES
47	YES	YES	YES	ND**	NS	NS	YES	YES	YES
48	YES	YES	YES	ND**	NS	NS	YES	YES	YES
49	YES	YES	NS	NS	NS	NS	YES	YES	NS
50	ND**	YES	YES	ND**	NS	NS	YES	YES	YES
Total "YES" samples, n (%)	38 (76%)	41 (82%)	40 (80%)	34 (68%)	33 (66%)	27 (54%)	44 (88%)	44 (88%)	37 (74%)

Supplementary Table 3. 16S rDNA-targeted primers and probes used in this study.

Target	Primers and Probes*	Sequence 5'-3'
All bacteria	F_Bact 1369F	CGG TGA ATA CGT TCC CGG
	R_Prok1492R	TAC GGC TAC CTT GTT ACG ACT T
	**P_TM 1389PR**	**6FAM-CTTGTACACACCGCCCGTC-TAMRA**
F. prausnitzii	Fpra 413F	TGTAAACTCCTGTTGTTGAGGAAGATAA
	Fpra 543R	GCGCTCCCTTTACACCCA
	**Fpra 454PR**	**6FAM-CAAGGAAGTGACGGCTAACTACGTGCCAG-TAMRA**
E. coli	E.coli 395 F	CATGCCGCGTGTATGAAGAA
	E.coli 491R	CGGGTAACGTCAATGAGCAAA
	**E.coli 468PR**	**6FAM-TATTAACTTTACTCCCTTCCTCCCCGCTGAA-TAMRA**
B. fragilis	B. Fragilis 44F	TCRGGAAGAAAGCTTGCT
	B. Fragilis 206R	CATCCTTTACCGGAATCCT
	**B. Fragilis 93PR**	**6FAM-ACACGTATCCAACCTGCCCTTTACTCG-TAMRA**
DNA IAC‡	IAC F	TACGGATGAGGAGGACAAAGGA
	IAC R	CACTTCgCTCTgATCCATTgg
	**IAC PR**	**VIC®-CGCCGCTATGGGCATCGCA-TAMRA**

**Probe sequences are in bold. P_TM 1389PR, E. coli 468PR , B. fragilis 93PR and Fpra 454PR probes were 5'-labelled with FAM™ (6-carboxyfluorescin) as the reporter dye, whereas the IAC probe was 5'-labeled with VIC®(6-carboxyrhodamine) as reporter dye to allow multiplex detection. TAMRA™ was used as quencher dye at the 3'-end for all the probes. ‡IAC, Internal Amplification Control; DNA IAC sequence (5'-3'): TACGGATGAGGAGGACAAAGGACGCCGCTATGGGCATCGCACCAATGGATCAGAGCGAAGTG*

Supplementary Table 4. Antibodies used for flow cytometry in mice.

Antigen	*Clone*	*Fluorochrome*	*Supplier*
**CD3ε**	145-2C11	APC-Cy7, BV421	BD bioscience
**CD4**	RM4-5	APC-A700	BD bioscience
**CD8α**	53-6.7	BV421	BioLegend
**CD11b**	M1/70	APC	BD bioscience
**CD11c**	HL3	AF-700	BD bioscience
**CD19**	1D3	BV421	BD bioscience
**CD25**	PC61	PE	BD bioscience
**CD44**	IM7	FITC	BD bioscience
**CD45**	30-F11	BV510	BD bioscience
**CD62L**	MEL-14	PerCP-Cy5.5	BD bioscience
**CD80**	16-10A1	PerCP-Cy5.5	BD bioscience
**CD86**	GL-1	PE	BD bioscience
**CD152**	UC10-4B9	PE-Cy7	BioLegend
**CD278**	7E.17G9	PE-Cy7	BD bioscience
**CD335**	29A1.4	BV421	BD bioscience
**FoxP3**	FJK-16s	APC	eBioscience
**I-Ab**	AF6-120.1	FITC	BD bioscience
**Ly6C**	AL-21	PE-Cy7	BD bioscience
**Ly6G**	1A8	APC-Cy7	BD bioscience

Supplementary Figures

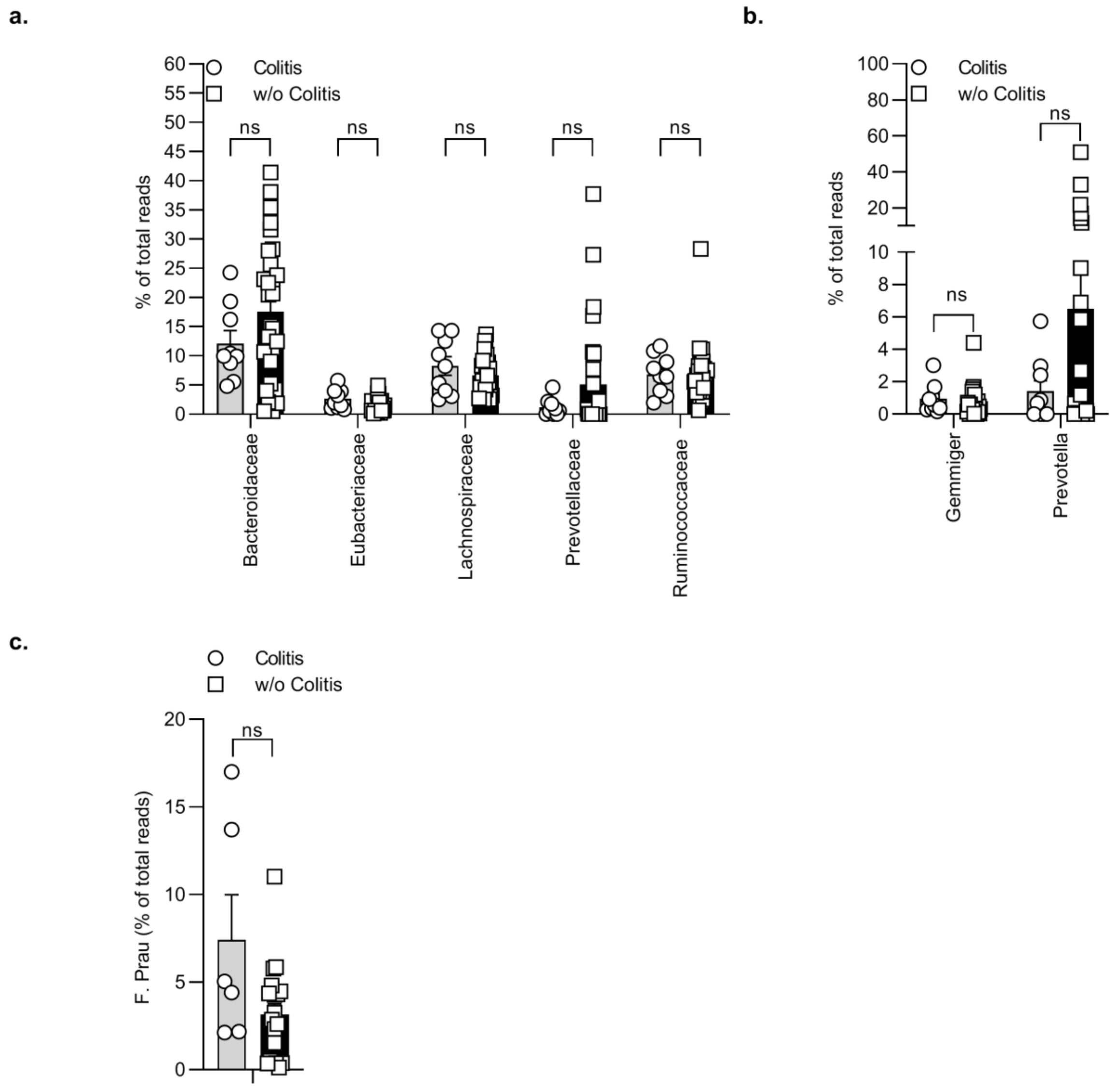

Supplementary Fig. 1. Gut microbiota composition at baseline and ipilimumab-induced colitis. **a.** Histograms (mean+/-SEM) of relative abundance of dominant family (*Bacteroidaceae, Lachnospiraceae, Eubacteriaceae, Prevotellaceae and Ruminococcaceae*), at baseline between patients prone (n=9) to or resistant (n=29) to ipilimumab-induced colitis **b.** Histograms (mean+/-SEM) of relative abundance of genus (*Lachnospiraceae*, *Gemmiger, Prevotella*) at baseline between patients prone (n=9) to or resistant (n=29) to ipilimumab-induced colitis and **c.** *Faecalibacterium prausnitzii* (NGS method did not identified F. *Prau* in all patients) at baseline between patients prone (n=6) to or resistant (n=19) to ipilimumab-induced colitis. Two-way ANOVA followed by Sidak's multiple comparisons (**a**, **b**) or Two-tailed Mann-Whitney (**c**) tests have been applied to assess significance and *p*-values or ns (not significant) are indicated on each graph.

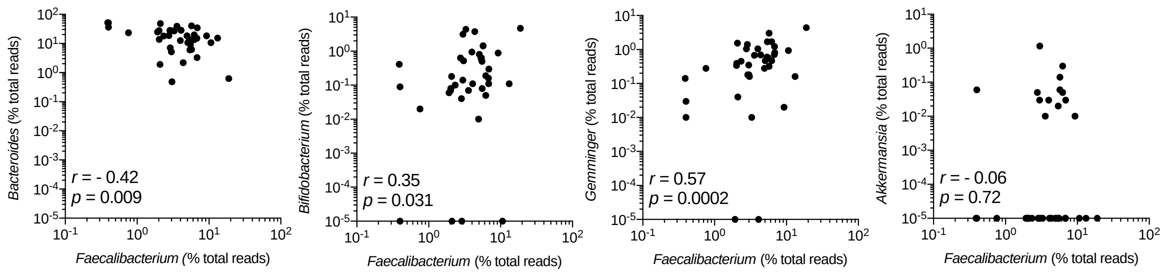

Supplementary Fig. 2. Correlations between *Faecalibacterium* and other genera. Data from 16S DNA analysis from patient's stools (n=38) were tested for correlation against *Faecalibacterium*. Pearson r correlation was used. *p* (two-tailed) and *r* are indicated on each graph.

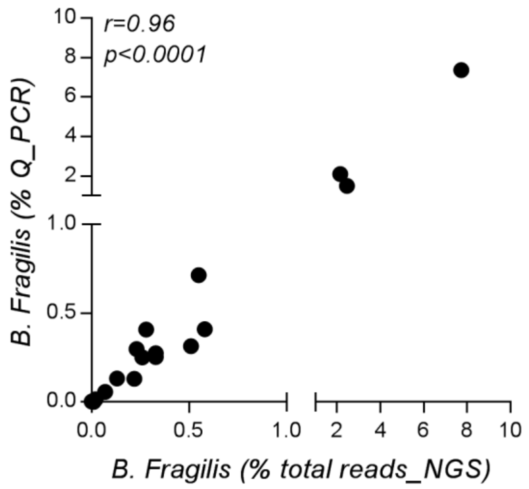

Supplementary Fig. 3. Spearman correlation between the relative abundance of *B. fragilis* (n=16; were both data were available) assessed by 16S metagenomic (% of total reads NGS) and Q-PCR (% Q-PCR) analyses on feces at baseline. *r* and *p* values are indicated on the graph.

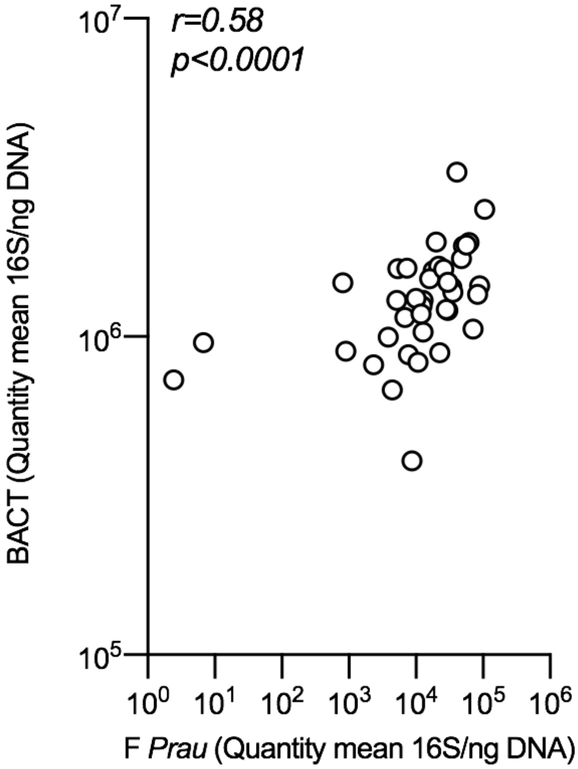

Supplementary Fig. 4. Correlations between *F. Prausnitzii* and quantity of total Bacteria in stool samples at baseline. Pearson correlation between the relative abundance of *F. praunitzii* (*F. Prau*) assessed by Q-PCR (quantity mean of 16S/ng DNA) analyses on feces at baseline and mean quantity of total bacterial DNA (quantity mean of 16S/ng DNA (BACT)) (n=38). r and *p* values are indicated on the graph.

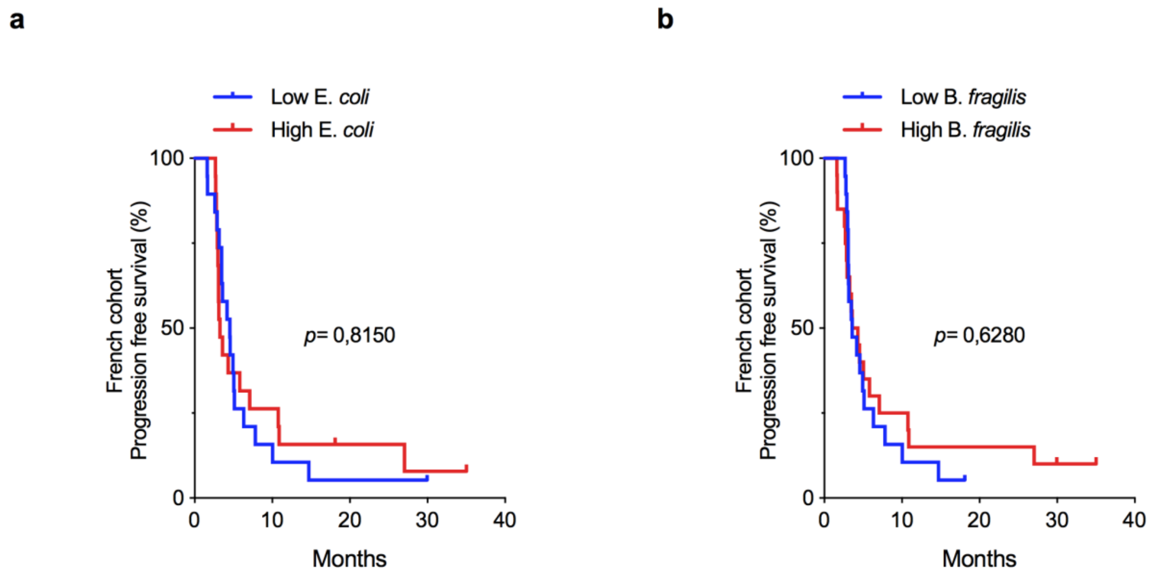

Supplementary Fig. 5. *E.coli* and *B. fragilis* are not associated with improved PFS in patients. Kaplan–Meier survival curves of PFS of patients from the French cohort (n=38) classified into two groups according to median of **a**, *E. coli* (quantity mean of 16S/ng DNA; Low vs high *E. coli*) and **b**, *B. fragilis* (quantity mean of 16S/ng DNA ; low vs high *B. fragilis*) at baseline. Log-rank (mantel-cox) test was used.

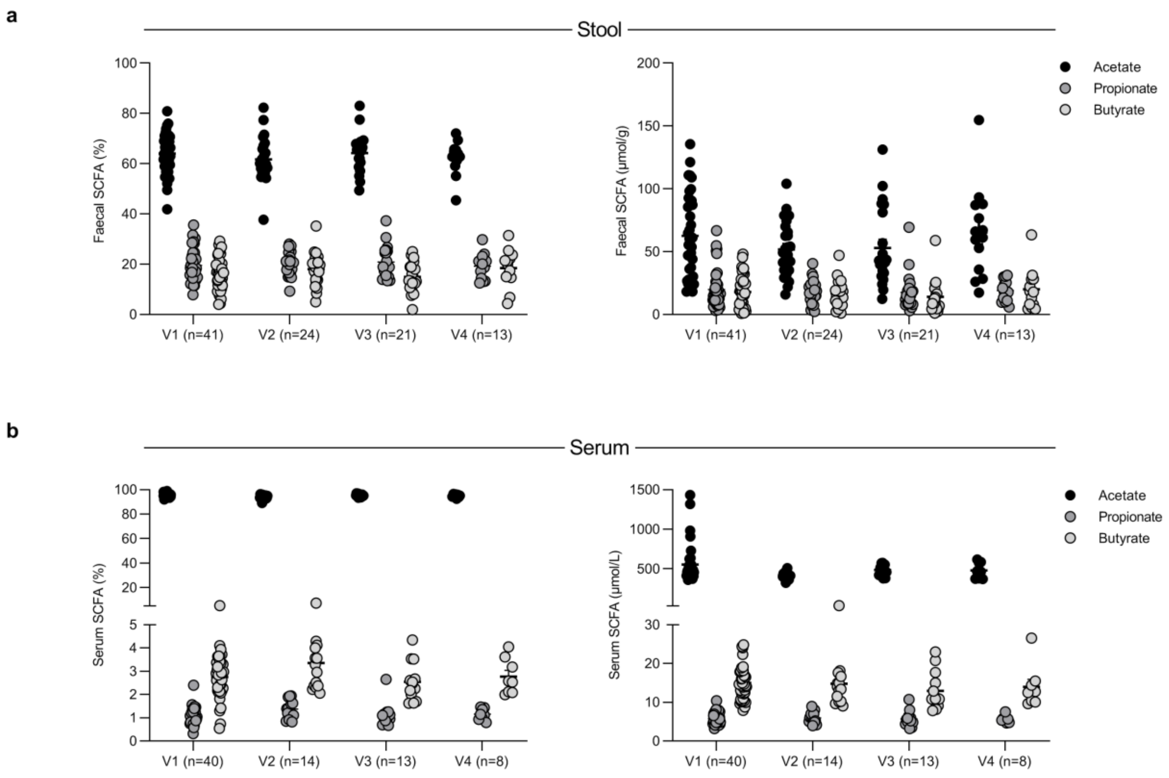

Supplementary Fig. 6. SCFA quantification in stool and sera in the French cohort.
a, Percentage (left panel) and concentration (right panel) of fecal SCFA. **b**, Percentage (left panel) and concentration (right panel) of serum SCFA. Each dot represents one patient, n are indicated on each graph.

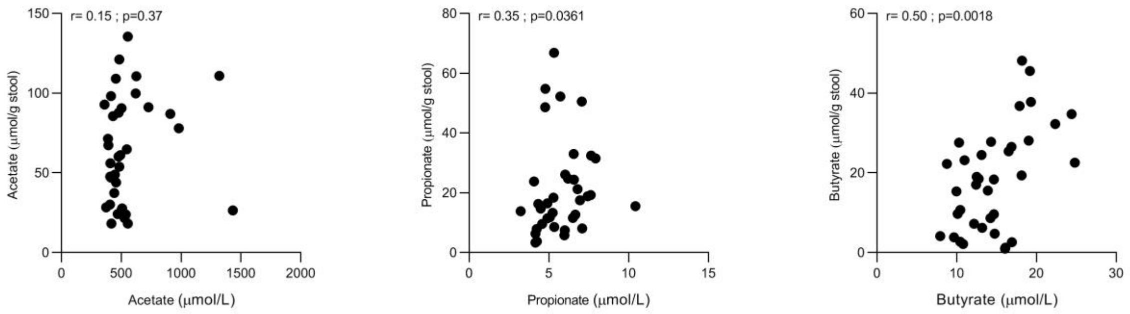

Supplementary Fig. 7. Correlations between fecal and serum SCFA in patients at baseline (V_1). Spearman correlations between concentrations of fecal and serum SCFA from the French cohort (n=36, where both pairs were available). Each dot represents one patient. *p* and *r* are indicated on each graphs.

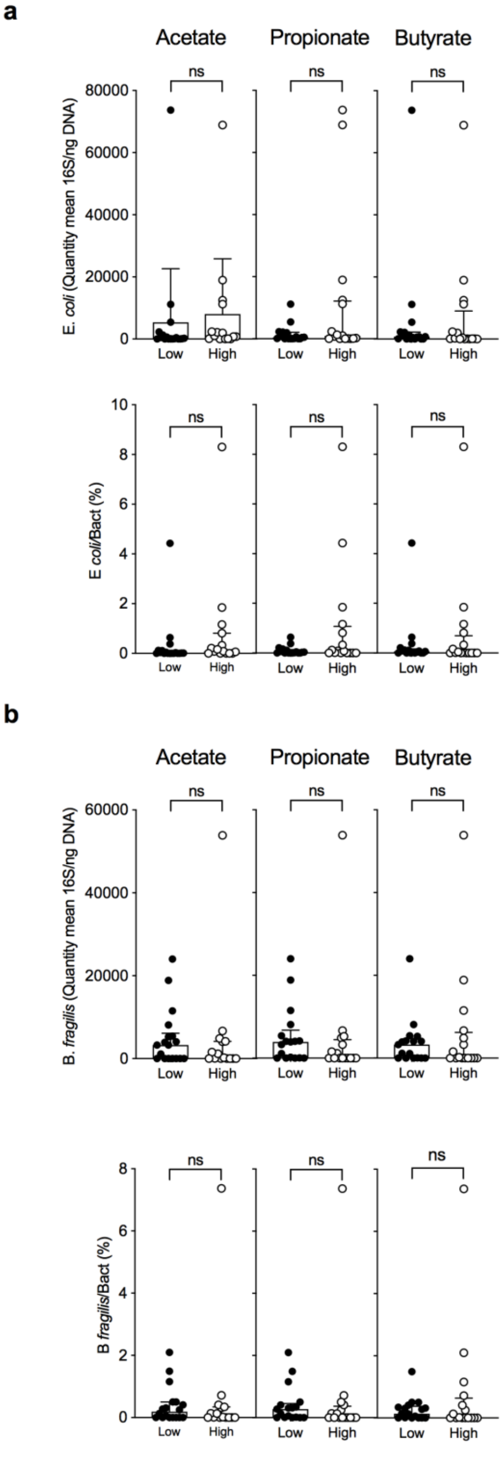

Supplementary Fig. 8. No association between *E. coli* or *B. fragilis* and serum concentrations of SCFA. a, Q-PCR analyses on feces at baseline for *E. coli (*16S *E. coli*/ng DNA (upper panel) and proportion (lower panel) according to the median concentrations of serum acetate, propionate and butyrate from the French cohort (n=33). **b**, As in (**a**) but for *B. fragilis*. Mann-Whitney (two-tailed) test has been applied to assess significance and *p*-values or ns (not significant) are indicated on the graph.

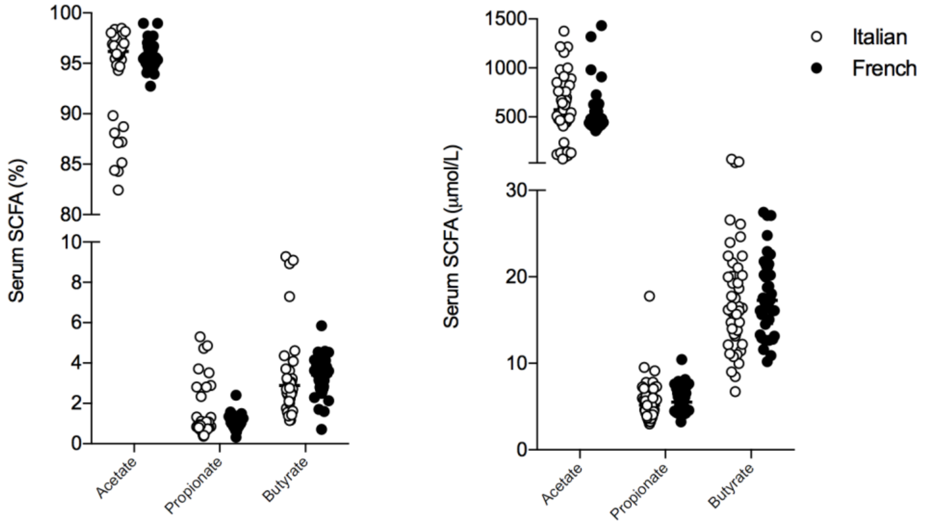

Supplementary Fig. 9. Comparison of serum SCFA in patients at baseline (V_1) between the French and the Italian cohort. Percentage (left panel) and concentrations (right panel) of serum acetate, propionate and butyrate between Italian cohort (white dots, n=45) and French cohort (black dots, n=40).

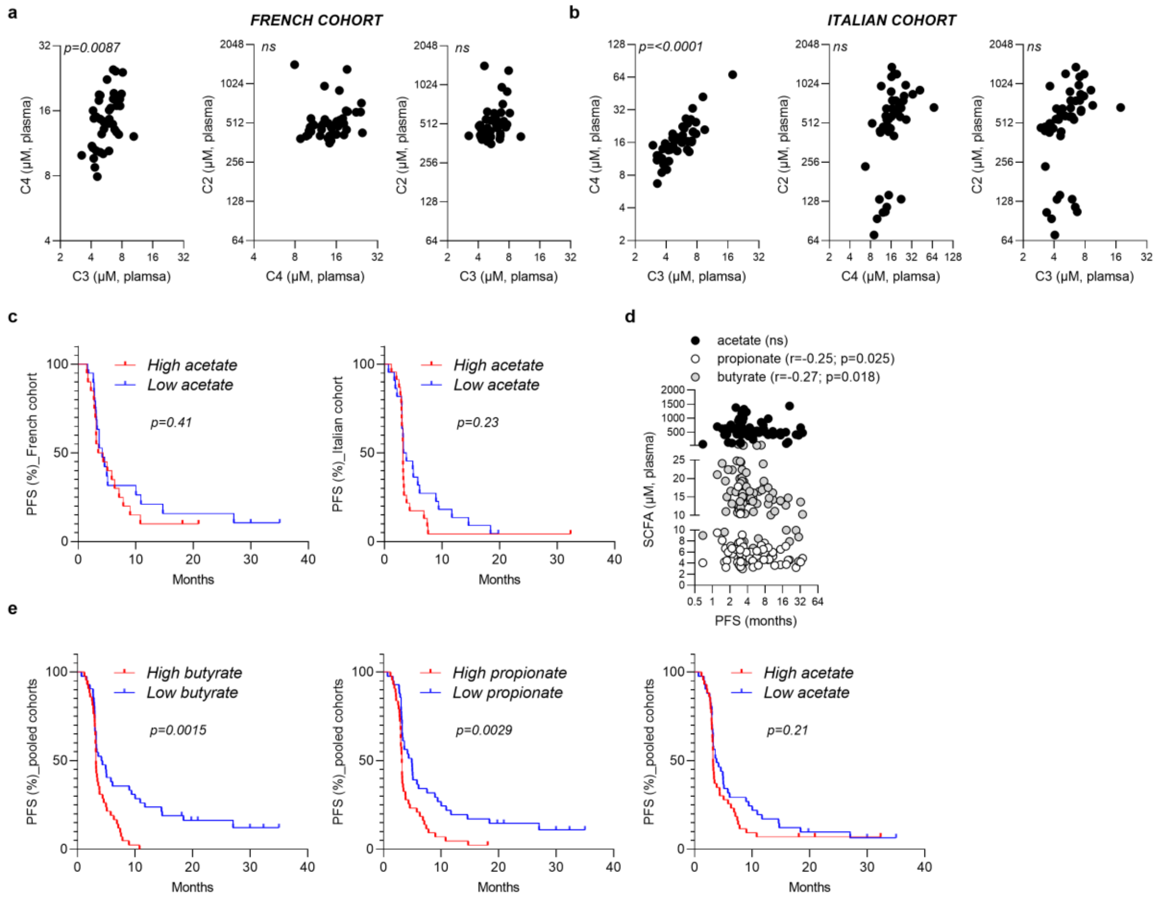

Supplementary Fig. 10. Correlations between serum SCFA in two independent cohorts of patients. a, Pearson correlations between serum of propionate (C3) and butyrate (C4, left panel), acetate (C2) and butyrate (middle panel) and acetate and propionate (right panel) in the French (n=40). **b,** As in (a) but for the Italian cohort (n=45). **c**. Kaplan–Meier survival curves of PFS according to the median value of serum acetate concentrations in French (left panel; n=40) and Italian (right panel; n=45) cohort. **d**, Pearson correlation between serum concentrations of acetate, butyrate and propionate and PFS pooling French and Italian cohorts (n=85); **e**. Kaplan–Meier survival curves of PFS according to the median value of serum butyrate (left panel), propionate (middle panel) and acetate (right panel) concentrations pooling French and Italian cohorts (n=85). Each dot represents one patient. Exact *p-values* and r are indicated on each graph; ns means non-significant.

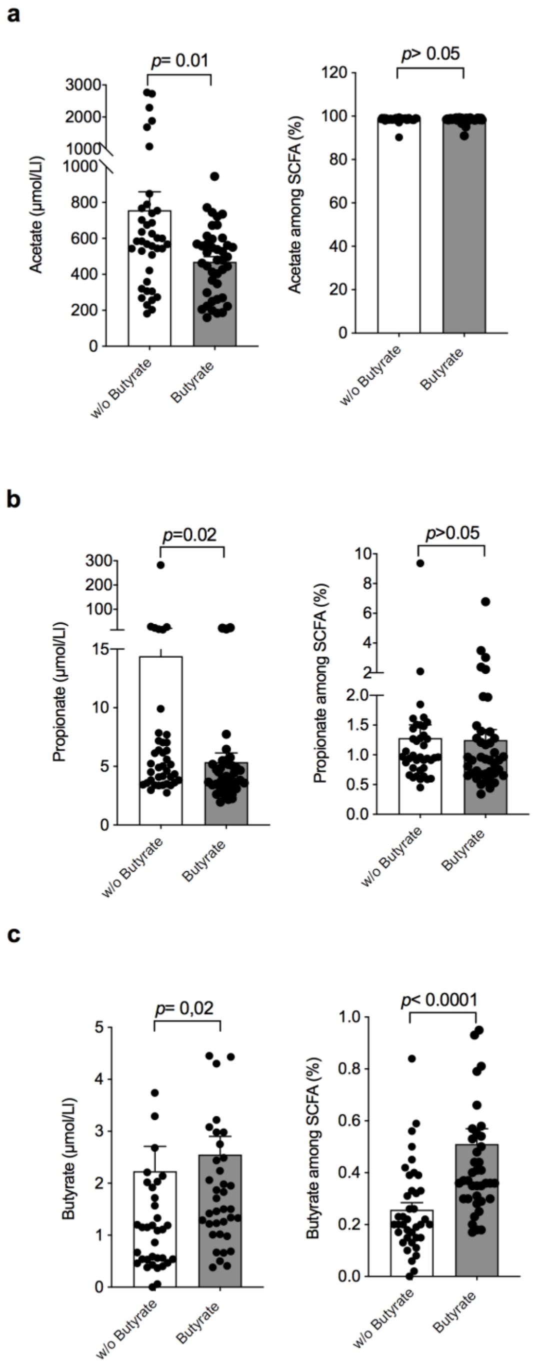

Supplementary Fig. 11. Concentrations of SCFA in mice serums. Mean (+/- SEM) concentrations (µmol/L; left panel) and percentage (%; right panel) of serum acetate (**a**), propionate (**b**) and butyrate (**c**) in mice drinking water (w/o Butyrate; n= 39 mice) and supplemented with sodium butyrate in drinking water (Butyrate; n= 41 mice). Each dot represent one mouse. *p*-values are indicated on each graph; two-tailed Mann–Whitney t-tests were used.

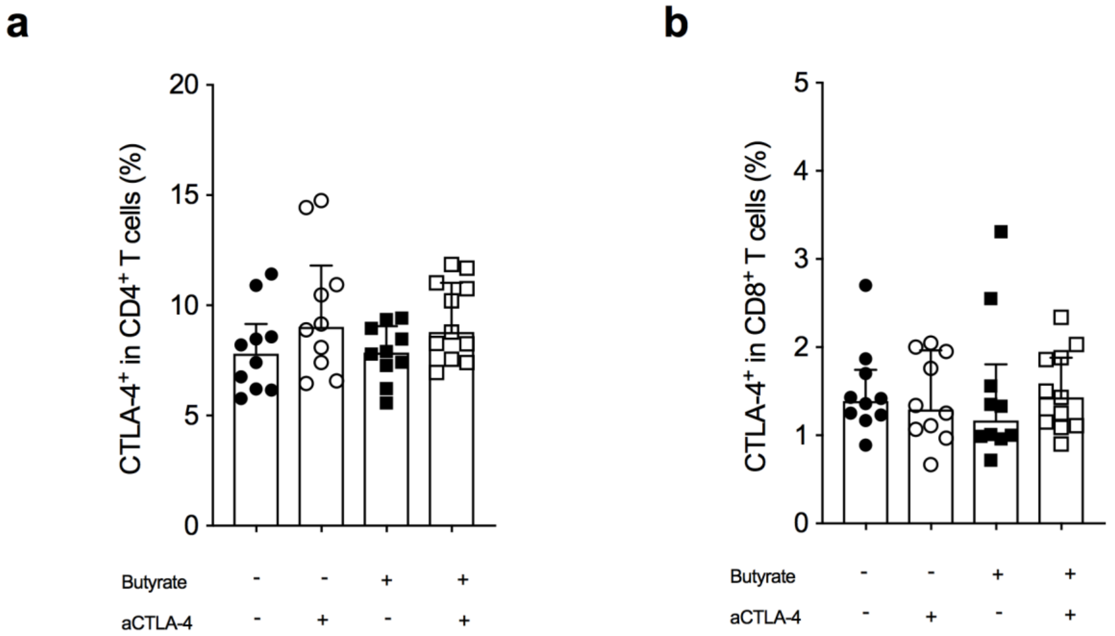

Supplementary Fig. 12. Intracellular expression of CTLA-4 among CD4$^+$ and CD8$^+$ T cells in tumor draining lymph nodes. Percentage of CTLA-4$^+$ among CD4$^+$ T cells (**a**) or CD8$^+$ T cells (**b**) in tDLN from pooled two independent experiments (n=10 or 11 (only for butyrate + aCTLA-4 group) mice). One-way ANOVA followed by Tukey's tests for multiple comparisons was used. No statistical differences were shown.

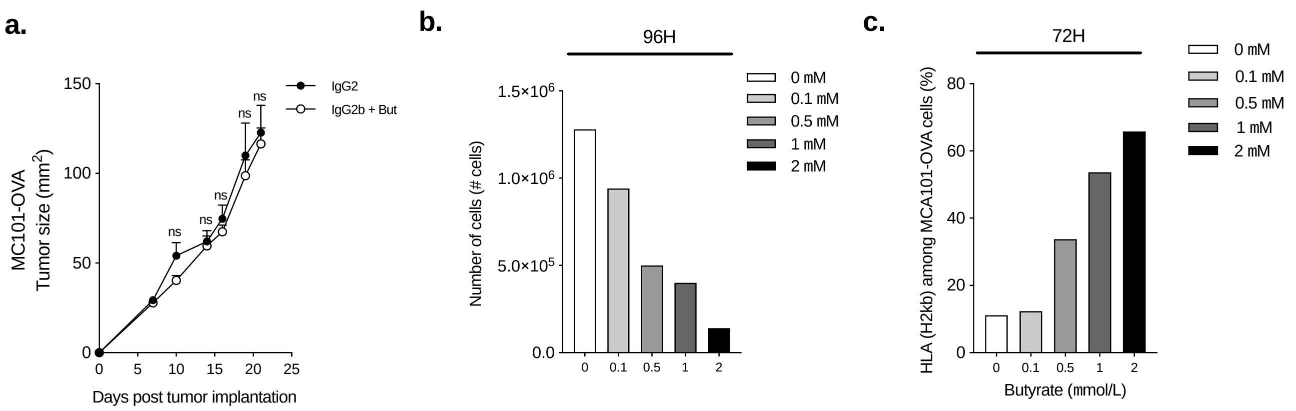

Supplementary Fig. 13: Sodium butyrate modified proliferation and H-2Kb expression of the MCA101-OVA cell line. a, Tumor growth of MCA101-OVA in mice treated with three injections of isotype control + PH-matched water (IgG2b) or isotype control + sodium butyrate (IgG2b + but). The graph depicts the mean ± SD of tumor sizes from one representative experiment out of 2 independent experiments (n = 10 mice per group). Two-way ANOVA followed by Sidak's multiple comparisons test was used. **b**, *In vitro* proliferation of MCA101-OVA cell line after 96h of culture in complete medium with escalating concentrations of sodium butyrate in vivo. **c**, Percentages of H-2Kb surface-expression after 72h of culture in complete medium with escalating concentrations of sodium butyrate in vivo. Mann–Whitney tests were used. ns, not-significant.

a

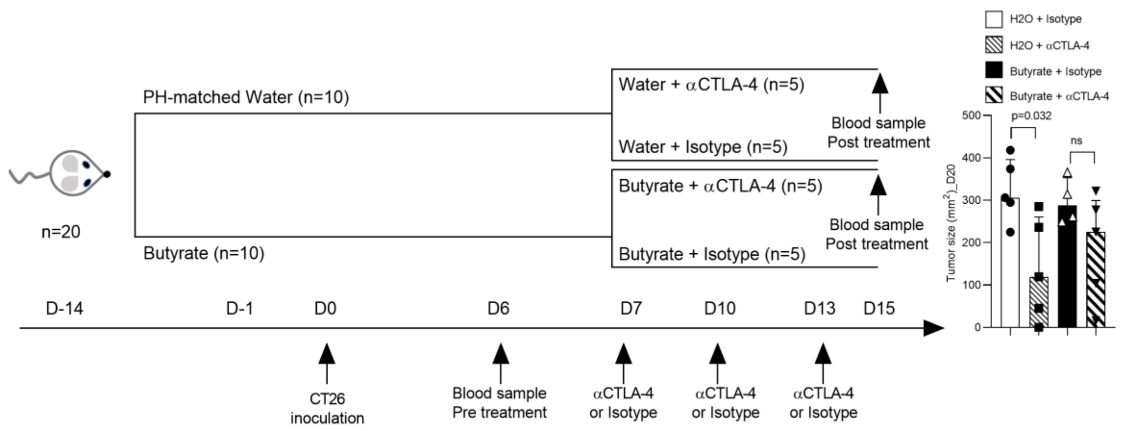

b

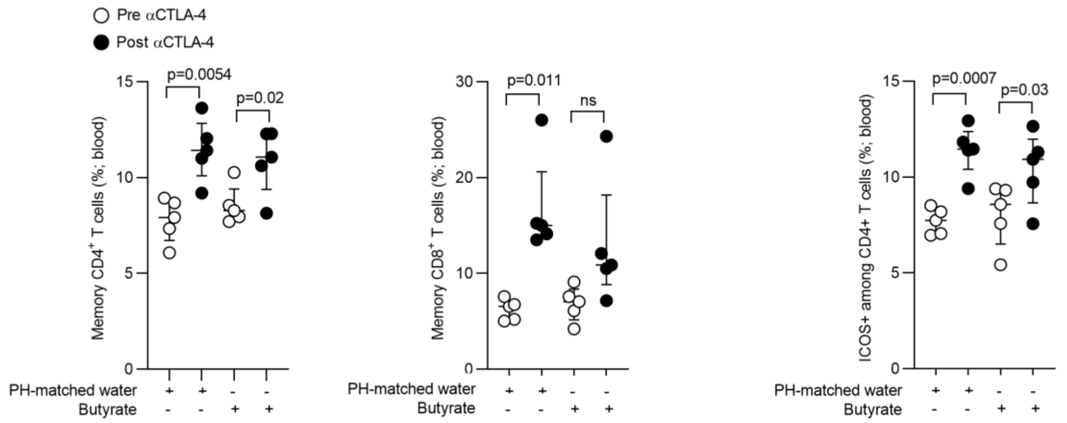

Supplementary Fig. 14. Longitudinal blood immune monitoring in mice. Mice were fed with butyrate (n=10) or PH-matched water (n=10) from day -14 (D-14), CT26 tumor cells were inoculated at day 0 (D0) in both groups and treatment started at day 7 (D7) with anti-CTLA-4 blocking mAbs (aCTLA-4, n=5) or its isotype control (Isotype, n=5) and was also performed at day 10 (D10) and day 13 (D13). Blood samples were performed at day 6 (D6) after tumor inoculation but before starting the treatments (pre-aCTLA-4) and at day 15, two days after the last injection of treatments (post-aCTLA-4). **a**, Left panel summarized the experimental settings of the longitudinal experiment and right panel represents the median tumor sizes (mm2 +/- SEM) at Day 20 in each group. **b**, Percentages (%) of memory $CD4^+$ T cells were monitored in fresh whole blood before (pre-aCTLA-4) and after aCTLA-4 treatment (post-aCTLA-4) (left panel); Percentages (%) of memory $CD8^+$ T cells were monitored in fresh whole blood before (pre-aCTLA-4) and after aCTLA-4 treatment (post-aCTLA-4) (middle panel); Percentages (%) of $ICOS^+$ $CD4^+$ T cells were monitored in fresh whole blood before (pre-aCTLA-4) and after aCTLA-4 treatment (post-aCTLA-4) (right panel). Each dot represents one mouse. Data are presented as median values with interquartile range. Exact p-values are indicated on each graph; Mann Whitney test was used (**a**, right panel) and paired t (two-tailed) tests were used (**b**). ns, not significant. Preliminary data from one experiment is shown.

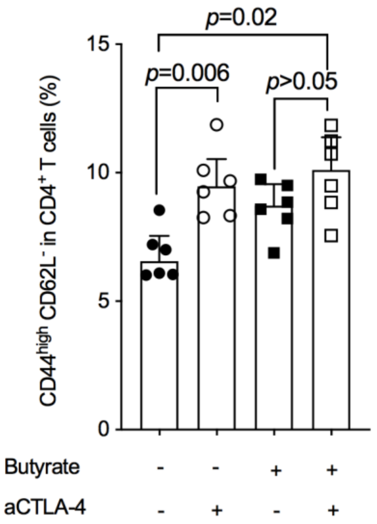

Supplementary Fig. 15. Comparison of the proportion of memory CD4$^+$ T cells in tDLN according to sodium butyrate supplementation. Percentage of central memory CD4$^+$ T cells (CD3$^+$CD4$^+$CD44^{hi}CD62L$^-$) in tumor draining lymph nodes (tDLN) of mice (n= 6 per group) drinking water (black and white circles) compared to mice supplemented in sodium butyrate (black and white squares). Each dot represents one mouse. Data are presented as median values with interquartile range. One-way ANOVA followed by Tukey's tests for multiple comparisons was used. Exact *p*-values are indicated on each graph.

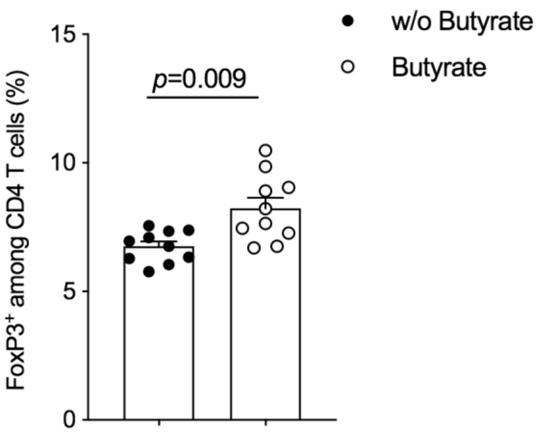

Supplementary Fig. 16. Comparison of the proportion of Treg cells in tDLN according to sodium butyrate enrichment. Percentage of Treg cells ($CD3^+CD4^+FoxP3^+$) in tDLN of mice (n= 10 per group) drinking water (black dots) compared to mice supplemented in sodium butyrate in drinking water (white dots). Each dot represents one mouse. Data are presented as mean values +/- SEM. Mann–Whitney t-tests were used (two-tailed). *p*-values are indicated on each graph.

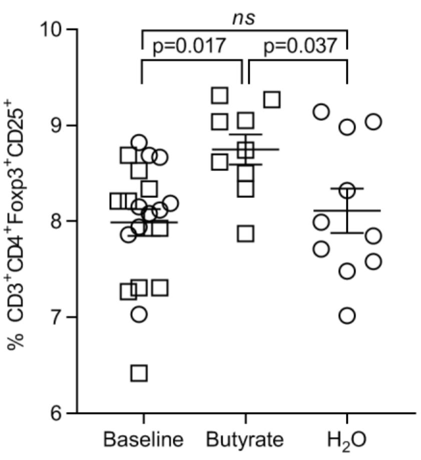

Supplementary Fig. 17. Comparison of the proportion of Treg cells in the blood. Percentage of Treg cells ($CD3^{+}CD4^{+}FoxP3^{+}$) in the blood of naïve mice at baseline (n= 20), after drinking water (H_2O, n=10; white circles) or drinking sodium butyrate in drinking water (Butyrate, n=10; white squares). Data are presented as means +/- SEM. Wilcoxon (baseline versus butyrate (white squares) and baseline versus H_2O (white circles) or Mann–Whitney (H_2O versus butyrate groups) tests were used (two-tailed).Exact p-values are indicated on the graph; ns, not significant.

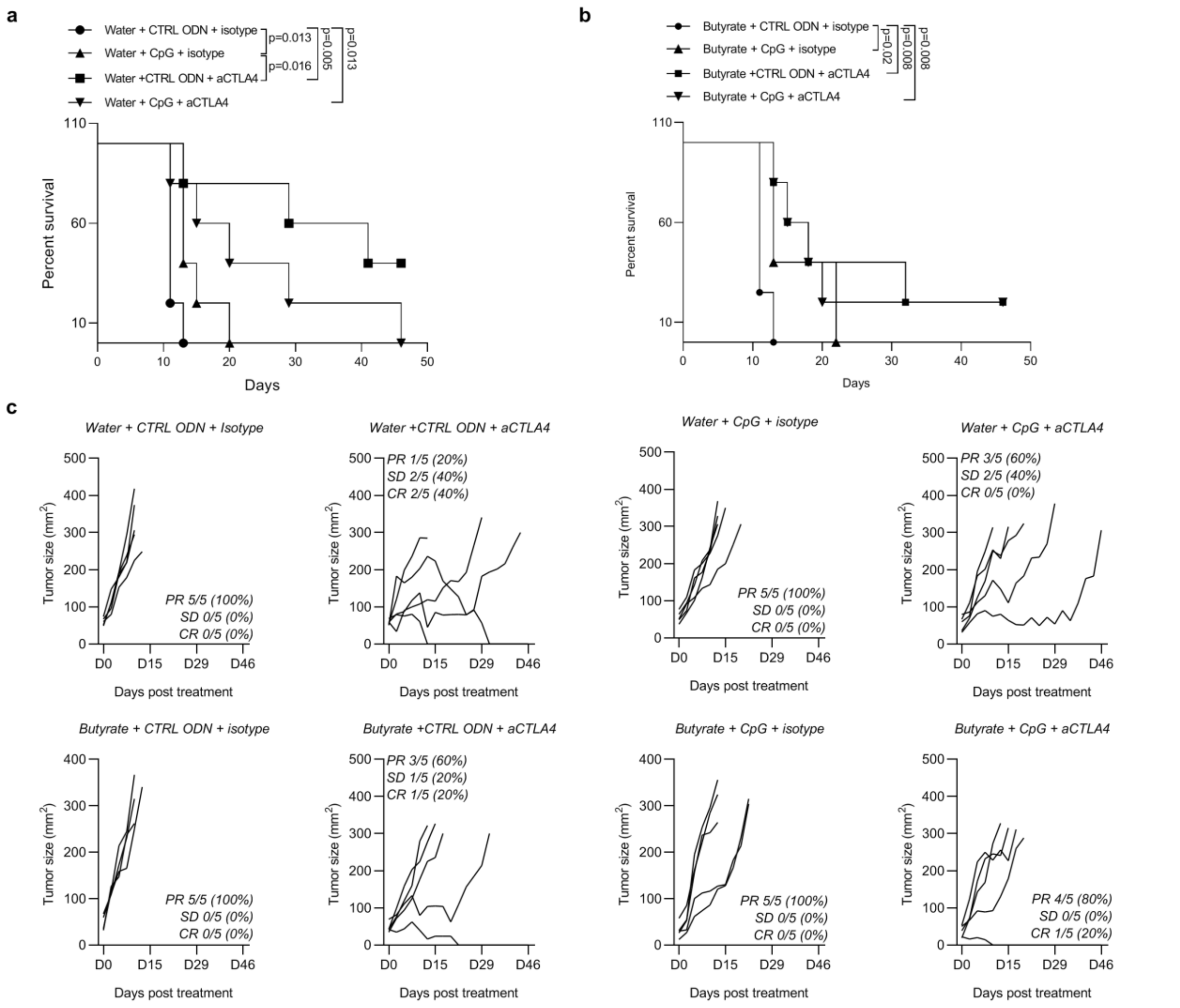

Supplementary Fig. 18. CpG agonists did not leverage butyrate + CTLA-4 treatment in mice. Twenty mice were treated 2 weeks before tumor inoculation and all along the experiment with sodium butyrate in the drinking water (n=10) at the concentrations of 100mM or pH-matched water (n=10) and changed every week. Two hundred thousand of CT26 tumor cells were inoculated subcutaneously (s.c) in the right flank of Balb/c mice fed with sodium butyrate in the drinking water (right panel) or PH-matched water (left panel) at day 0 . Mice received intraperitoneal (i.p) injections of 100 µg of anti-CTLA-4 (aCTLA-4, n=5) or its isotype control (Isotype, n=5) at D7, D10 and D13. Mice received subcutaneously (s.c) injections of 30 µg of CpG (CpG, n=5) or ODN control (CTRL ODN, n=5) at D7, D10 and D13. Tumor growth was measured three times a week. Mice were euthanized when the tumor size was ≥ 300 mm^2 or boundary points were reached. **a,** Percent survival of mice with PH-matched water (left panel); ; Log-rank (Mantel-Cox) test was used. **b,** Percent survival of mice with butyrate (right panel); Log-rank (Mantel-Cox) test was used. **c,** tumor size over time after tumor inoculation in each group. Each line represents one mouse. Progressive disease (PD), stable disease (SD) and complete responses (CR) are indicated on each graph. Exact *p-values* are indicated on the graphs otherwise not-significant.

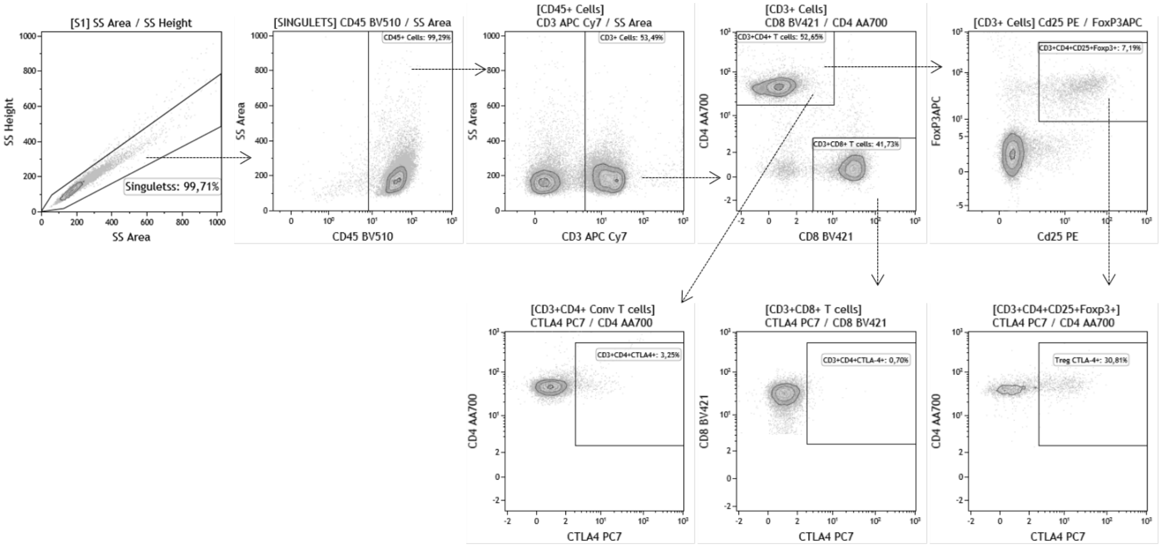

Supplementary Fig.19. Representative gating strategy in mice for Tregs and CTLA-4+ T cells

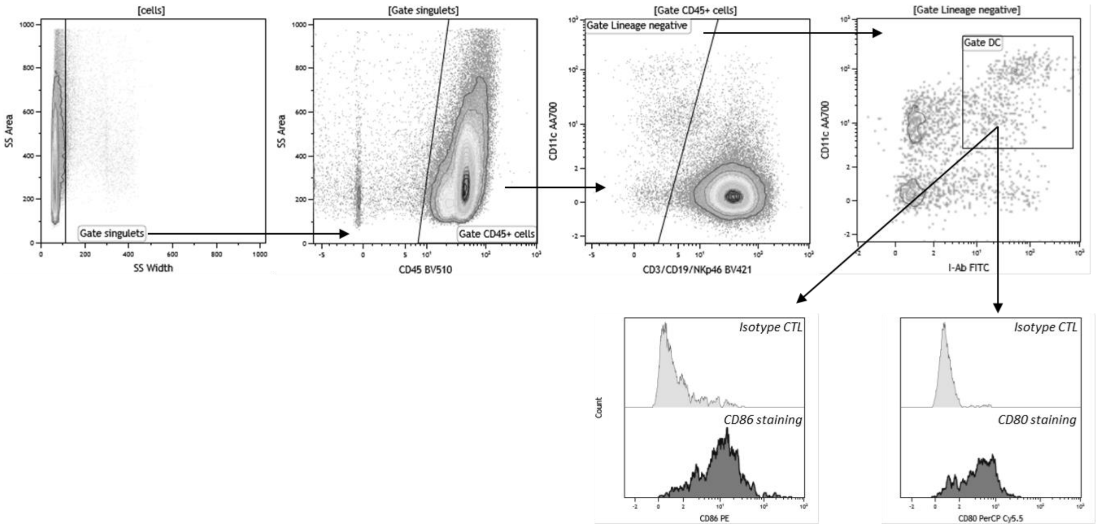

Supplementary Fig.20. Representative gating strategy in mice for dendritic cells.

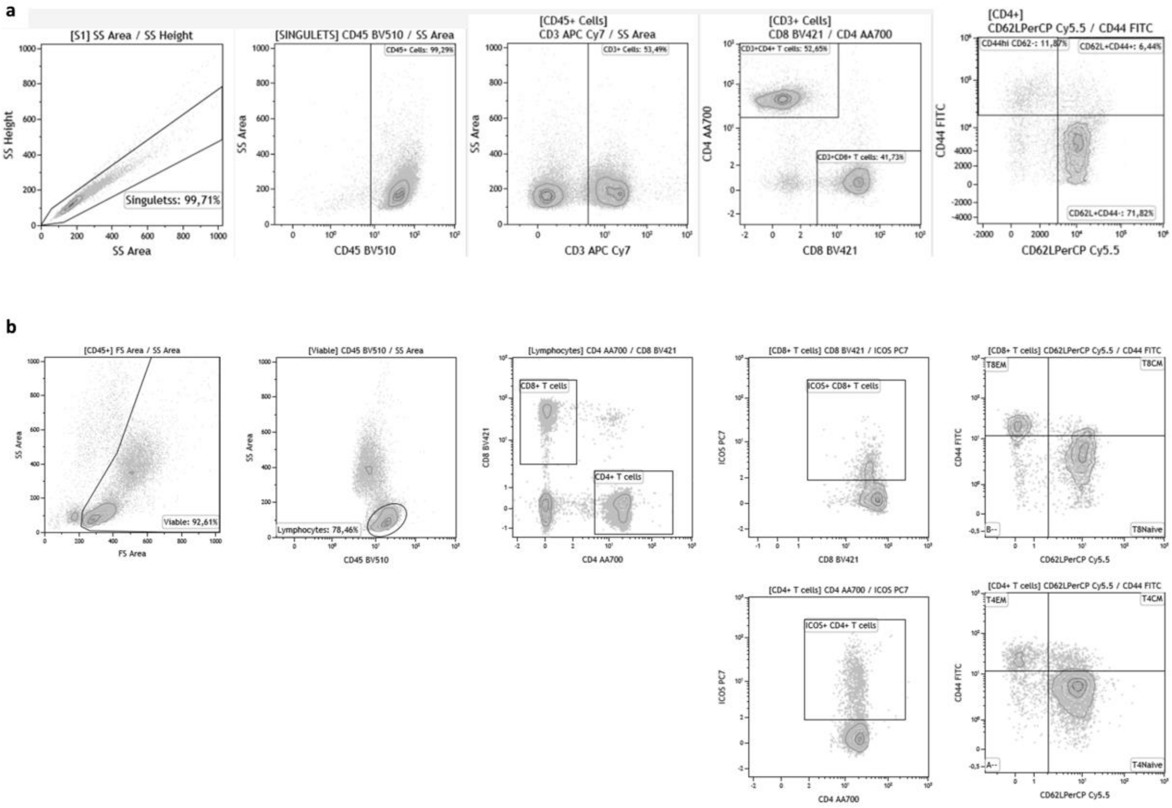

Supplementary Fig.21. a. Representative gating strategy in mice CD44^{hi}CD62L^{-}CD4^{+} T cells in lymph nodes .**b.** Representative gating strategy in mice CD44^{hi}CD62L^{-}CD4^{+} T cells and ICOS^{+} T cells in the blood.

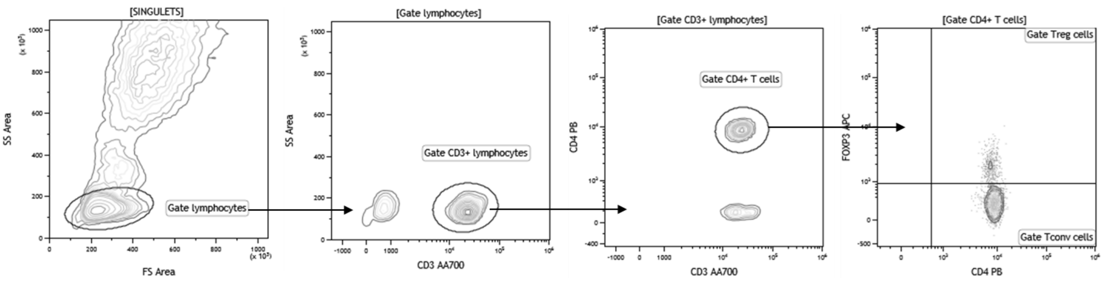

Supplementary Fig.22. Representative gating strategy in Human whole blood for Foxp3 staining after permeabilization.

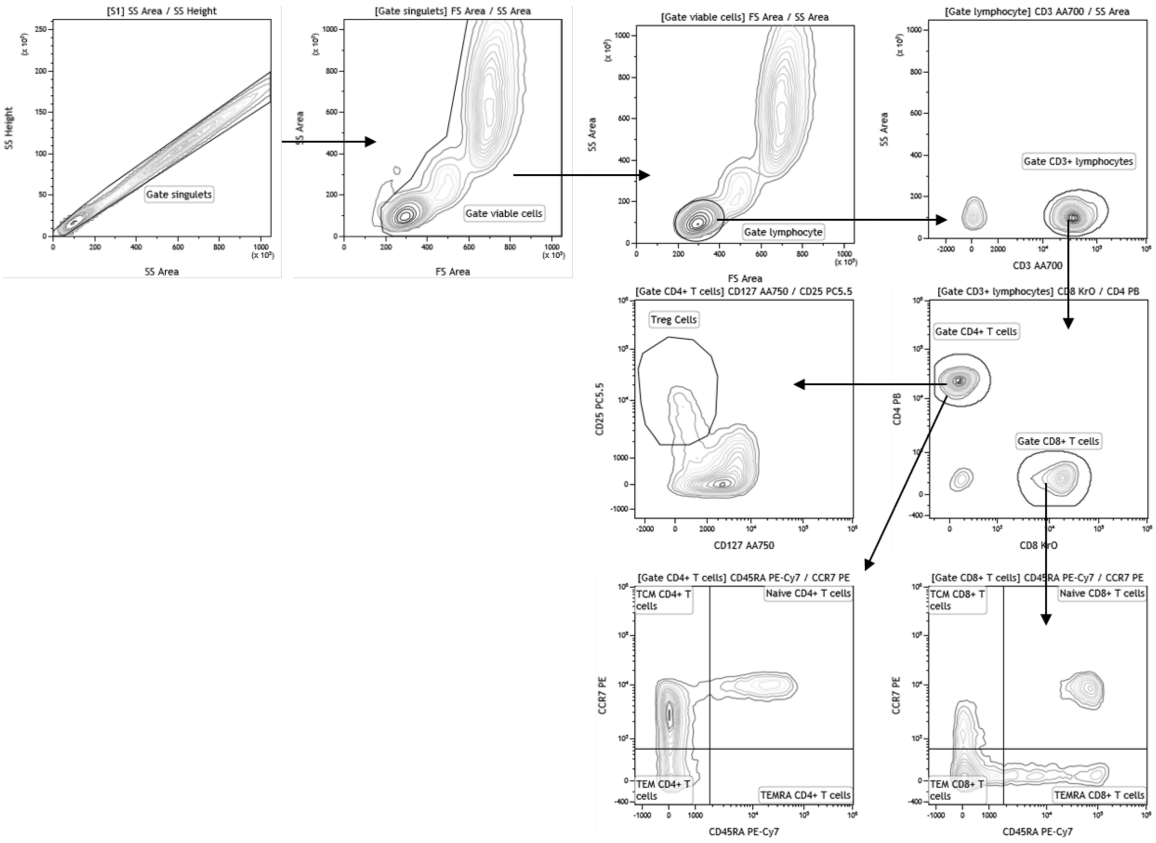

Supplementary Fig.23. Representative gating strategy in Human whole blood sample
