# Supplementary material for: Systemic short chain fatty acids limit antitumor effect of CTLA-4 blockade in hosts with cancer
Source: Nat Commun. 2020 May 1;11:2168. doi: 10.1038/s41467-020-16079-x (PMC7195489; doi:10.1038/s41467-020-16079-x)
Supplement: Supplementary file 3 — Reporting Summary [file 41467_2020_16079_MOESM3_ESM.pdf]

natureresearch

Corresponding author(s): Nathalie CHAPUT

Last updated by author(s): 15/01/2020

# Reporting Summary

Nature Research wishes to improve the reproducibility of the work that we publish. This form provides structure for consistency and transparency in reporting. For further information on Nature Research policies, see Authors & Referees and the Editorial Policy Checklist .

## Statistics

For all statistical analyses, confirm that the following items are present in the figure legend, table legend, main text, or Methods section.

| n/a | Confirmed | |
|---|---|---|
| ☐ | ☒ | The exact sample size (*n*) for each experimental group/condition, given as a discrete number and unit of measurement |
| ☐ | ☒ | A statement on whether measurements were taken from distinct samples or whether the same sample was measured repeatedly |
| ☐ | ☒ | The statistical test(s) used AND whether they are one- or two-sided<br>*Only common tests should be described solely by name; describe more complex techniques in the Methods section.* |
| ☐ | ☒ | A description of all covariates tested |
| ☐ | ☒ | A description of any assumptions or corrections, such as tests of normality and adjustment for multiple comparisons |
| ☐ | ☒ | A full description of the statistical parameters including central tendency (e.g. means) or other basic estimates (e.g. regression coefficient) AND variation (e.g. standard deviation) or associated estimates of uncertainty (e.g. confidence intervals) |
| ☒ | ☐ | For null hypothesis testing, the test statistic (e.g. *F*, *t*, *r*) with confidence intervals, effect sizes, degrees of freedom and *P* value noted<br>*Give P values as exact values whenever suitable.* |
| ☒ | ☐ | For Bayesian analysis, information on the choice of priors and Markov chain Monte Carlo settings |
| ☒ | ☐ | For hierarchical and complex designs, identification of the appropriate level for tests and full reporting of outcomes |
| ☒ | ☐ | Estimates of effect sizes (e.g. Cohen's *d*, Pearson's *r*), indicating how they were calculated |

*Our web collection on statistics for biologists contains articles on many of the points above.*

## Software and code

Policy information about availability of computer code

| | |
|---|---|
| Data collection | For Cytometry acquisition data was realized with Kaluza v2.1.1, Beckman Coulter. For cytokine analyses the MAGPIX® System from Luminex was used. For SCFA quantification, the system control and data acquisition were performed using MassLynx® software (v4.1, Waters, Milford, MA, USA). Ion S5 Torrent Sequencing Analysis : Base calling and run demultiplexing were performed by using Torrent_Suite Software, version 4.0.2. All sequencing data were exported in Ion Reporter Software, version 5.6 with Metagenomic workflow which detects the bacterial diversity from a metagenomics sample from Ion semiconductor reads from the Ion 16S Metagenomics Kit. |
| Data analysis | Cytometry data were realized with Kaluza v2.1.1, Beckman Coulter. Cytokine analyses was realized with xPONENT® SOFTWARE, basic xPONENT software package. For sCTLA-4 ELISA determination of concentrations were assessed with the iMark™ Microplate Absorbance Reader controlled by a PC with Microplate Manager® 6 Software (Bio-Rad). statistical analyses were performed using the Prism 7.0 (GraphPad) Software and PROC lifetest of SAS software, Version 9.4 (SAS Institute, Inc., Cary, NC). t-distributed stochastic neighbor embedding (t-SNE) algorithm using the online R software (version 3.5.0, cytofkit package), visualized using the online Morpheus software and robust Z-score normalization (https://software.broadinstitute.org/morpheus/). |

For manuscripts utilizing custom algorithms or software that are central to the research but not yet described in published literature, software must be made available to editors/reviewers. We strongly encourage code deposition in a community repository (e.g. GitHub). See the Nature Research guidelines for submitting code & software for further information.

## Data

Policy information about availability of data

All manuscripts must include a data availability statement. This statement should provide the following information, where applicable:

- Accession codes, unique identifiers, or web links for publicly available datasets
- A list of figures that have associated raw data
- A description of any restrictions on data availability

The data that support the findings of this study are available from the first and the corresponding authors upon reasonable request.

# Field-specific reporting

Please select the one below that is the best fit for your research. If you are not sure, read the appropriate sections before making your selection.

☒ Life sciences ☐ Behavioural & social sciences ☐ Ecological, evolutionary & environmental sciences

For a reference copy of the document with all sections, see nature.com/documents/nr-reporting-summary-flat.pdf

# Life sciences study design

All studies must disclose on these points even when the disclosure is negative.

| | |
|---|---|
| Sample size | For mice data, we used 6 to 10 mice per group in order to be able to conclude to a notable or statistical effect. Experiments were repeated at least 2 times to ensure the reproducibility and validity of the results from one experiment to another. If the number of animals in at least one group was less than 30, non-parametric statistical tests were used. If the number of animals was equal to or greater than 30 in all groups then parametric statistical tests were used. The number of animals is scrupulously controlled in order to respect the ethical rules. For Human data, experiments were performed in a controlled and non-blinded manner. No randomization and no sample size-calculation was performed prior this study because of the observational nature of the study. The French cohort is considered as a discovery set and the Italian cohort as validation cohort to confirm that SCFA could be linked to clinical outcomes. |
| Data exclusions | No existing data were excluded from analyses |
| Replication | In mice; experiments were reproduced at least 2 times, pooled data or one representative experiments are shown in the manuscript.<br>For human data, a discovery (French Cohort) and a validation cohorte (Italian Cohort). |
| Randomization | Not relevant for our study |
| Blinding | Blinding was not relevant for this study. |

# Reporting for specific materials, systems and methods

We require information from authors about some types of materials, experimental systems and methods used in many studies. Here, indicate whether each material, system or method listed is relevant to your study. If you are not sure if a list item applies to your research, read the appropriate section before selecting a response.

### Materials & experimental systems

| n/a | Involved in the study | |
|---|---|---|
| ☐ | ☒ | Antibodies |
| ☐ | ☒ | Eukaryotic cell lines |
| ☐ | ☐ | Palaeontology |
| ☐ | ☒ | Animals and other organisms |
| ☐ | ☒ | Human research participants |
| ☐ | ☒ | Clinical data |

### Methods

| n/a | Involved in the study | |
|---|---|---|
| ☐ | ☐ | ChIP-seq |
| ☐ | ☒ | Flow cytometry |
| ☐ | ☐ | MRI-based neuroimaging |

## Antibodies

| | |
|---|---|
| Antibodies used | For Human cells, antibodies for flow cytometry were previously published by our team and are discribed in "Pitoiset, F. et al. Deep phenotyping of immune cell populations by optimized and standardized flow cytometry analyses. Cytom. Part J. Int. Soc. Anal. Cytol. 93, 793–802 (2018)."<br>For mice, antibodies used for flow cytometry are described "Supplementary Table 3. Antibodies used for flow cytometry in mice" |
| Validation | For human data antibodies used were validated and previously published "Pitoiset, F. et al. Deep phenotyping of immune cell populations by optimized and standardized flow cytometry analyses. Cytom. Part J. Int. Soc. Anal. Cytol. 93, 793–802 (2018)." |

For mice flow cytometry panels, all combination of antibodies were validated with FMO; for tricky staining we used isotype controls.

## Eukaryotic cell lines

Policy information about cell lines

| | |
|---|---|
| Cell line source(s) | Mouse colon carcinoma MC38 (previously reported in Tanikawa T et al. Cancer Res. 2012;72(2):420–429) and CT26 (American Type Culture Collection (ATCC, Manassas, VA) and fibrosarcoma MCA101OVA (kindly given by C. Sedlik, previously reported in Zeelenberg IS et al. Cancer Res. 2008 Feb 15; 68(4):1228-35.). All this are in the methods of the manuscript. |
| Authentication | None of the cell lines used were authenticated |
| Mycoplasma contamination | Cell lines were tested negative for mycoplasma (MycoProbe® Mycoplasma Detection Kit, R&D systems, Minneapolis, MN). |
| Commonly misidentified lines (See ICLAC register) | Not applicable |

## Palaeontology

| | |
|---|---|
| Specimen provenance | Not applicable |
| Specimen deposition | Not applicable |
| Dating methods | Not applicable |

☐ Tick this box to confirm that the raw and calibrated dates are available in the paper or in Supplementary Information.

## Animals and other organisms

Policy information about studies involving animals; ARRIVE guidelines recommended for reporting animal research

| | |
|---|---|
| Laboratory animals | C57Bl/6 and BALB/c mice were purchased from Harlan Laboratories (Gannat, France) between 8 and 14 weeks of age. |
| Wild animals | Not applicable, the study did not involved wild animal |
| Field-collected samples | Not applicable, the study did not involved samples collected from the field |
| Ethics oversight | All animal experiments were carried out in accordance with French and European laws and regulations and approved by the French Animal Experimentation Ethics Committee n°26 (02004.02). |

Note that full information on the approval of the study protocol must also be provided in the manuscript.

## Human research participants

Policy information about studies involving human research participants

| | |
|---|---|
| Population characteristics | Population caracteristics are described in supplemental table 1 of the manuscript |
| Recruitment | Forty French patients and forty-five Italian patients with metastatic melanoma treated with ipilimumab were prospectively enrolled at Gustave Roussy Cancer Campus (Villejuif) between March 2013 and December 2014 and at Instituto Nazionale Tumori Fondazione G. Pascale (Napoli) between July 2014 and March 2016 respectively. Patients were informed of the study and consented to participate. French patients had a pre-specified clinical workup; feces and blood were collected at baseline (V1), prior to each ipilimumab infusion (V2, V3, V4). |
| Ethics oversight | Immune monitoring in whole blood of patients was approved by the Kremlin Bicêtre Hospital Ethics Committee (SC12-018; ID-RCB-2012-A01496-37) and the Declaration of Helsinki protocols were followed. Patients provided their written informed consent to participate in these studies prior to inclusion in the study. |

Note that full information on the approval of the study protocol must also be provided in the manuscript.

## Clinical data

Policy information about clinical studies

All manuscripts should comply with the ICMJEguidelines for publication of clinical research and a completedCONSORT checklist must be included with all submissions.

| | |
|---|---|
| Clinical trial registration | Not applicable |
| Study protocol | Not applicable |

| | |
|---|---|
| Data collection | Not applicable |
| Outcomes | Not applicable |

# ChIP-seq

## Data deposition

☐ Confirm that both raw and final processed data have been deposited in a public database such as GEO.

☐ Confirm that you have deposited or provided access to graph files (e.g. BED files) for the called peaks.

| | |
|---|---|
| Data access links<br>*May remain private before publication.* | Not applicable |
| Files in database submission | Not applicable |
| Genome browser session<br>(e.g. UCSC) | Not applicable |

## Methodology

| | |
|---|---|
| Replicates | Not applicable |
| Sequencing depth | Not applicable |
| Antibodies | Not applicable |
| Peak calling parameters | Not applicable |
| Data quality | Not applicable |
| Software | Not applicable |

# Flow Cytometry

## Plots

Confirm that:

☒ The axis labels state the marker and fluorochrome used (e.g. CD4-FITC).

☒ The axis scales are clearly visible. Include numbers along axes only for bottom left plot of group (a 'group' is an analysis of identical markers).

☒ All plots are contour plots with outliers or pseudocolor plots.

☒ A numerical value for number of cells or percentage (with statistics) is provided.

## Methodology

| | |
|---|---|
| Sample preparation | Sample preparations are described in materials and methods section for mice and human data. |
| Instrument | Gallios Cytometer (Beckman Coulter) described in materials and methods section |
| Software | Cytometry data acquisition and analyses were done with Kaluza v2.1.1 from Beckman Coulter. |
| Cell population abundance | Not cell sorting was realized in this study |
| Gating strategy | Gating strategy are shown in supplementary figures 19 to 23. |

☒ Tick this box to confirm that a figure exemplifying the gating strategy is provided in the Supplementary Information.

# Magnetic resonance imaging

## Experimental design

| | |
|---|---|
| Design type | Not applicable |
| Design specifications | Not applicable |
| Behavioral performance measures | Not applicable |

## Acquisition

| | |
|---|---|
| Imaging type(s) | Not applicable |
| Field strength | Not applicable |
| Sequence & imaging parameters | Not applicable |
| Area of acquisition | *State whether a whole brain scan was used OR define the area of acquisition, describing how the region was determined.* |

Diffusion MRI ☐ Used ☒ Not used

## Preprocessing

| | |
|---|---|
| Preprocessing software | Not applicable |
| Normalization | Not applicable |
| Normalization template | Not applicable |
| Noise and artifact removal | Not applicable |
| Volume censoring | Not applicable |

## Statistical modeling & inference

| | |
|---|---|
| Model type and settings | Not applicable |
| Effect(s) tested | Not applicable |

Specify type of analysis: ☐ Whole brain ☐ ROI-based ☐ Both

| | |
|---|---|
| Statistic type for inference (See Eklund et al. 2016) | Not applicable |
| Correction | Not applicable |

## Models & analysis

| n/a | Involved in the study |
|---|---|
| ☒ | ☐ Functional and/or effective connectivity |
| ☒ | ☐ Graph analysis |
| ☒ | ☐ Multivariate modeling or predictive analysis |
